# Genetic Diversity of Invasive *Spartina alterniflora* Loisel. (Poaceae) Introduced Unintentionally Into Japan and Its Invasion Pathway

**DOI:** 10.3389/fpls.2020.556039

**Published:** 2020-09-07

**Authors:** Yu Maebara, Masanori Tamaoki, Yuka Iguchi, Naoyuki Nakahama, Takaaki Hanai, Atsushi Nishino, Daisuke Hayasaka

**Affiliations:** ^1^ Graduate School of Agriculture, Kindai University, Nara, Japan; ^2^ Fukushima Branch, National Institute for Environmental Studies (NIES), Miharu, Japan; ^3^ Institute of Natural and Environmental Sciences, University of Hyogo, Sanda, Japan; ^4^ Division of Ecological Restoration, The Museum of Nature and Human Activities, Hyogo, Sanda, Japan; ^5^ Japanese Network for Prevention Spartinas Spreading (JNPS), Nagoya, Japan

**Keywords:** biological invasion, chloroplast DNA, founder effect, genetic structure, microsatellite, secondary introduction, smooth cordgrass, trade history

## Abstract

Among invasive species, aquatic plants pose serious threats to local biodiversity and ecosystem functions. *Spartina alterniflora* Loisel. (Poaceae), native to the eastern United States, was introduced unintentionally into Japan (Aichi and Kumamoto Prefectures) at around 2010. This invasive species could easily and rapidly spread to estuarine areas of Japan *via* vigorous trade and transport, making the prediction of its future invasion necessary. Here, the distribution and structure of the genetic variation of *S. alterniflora* in Japan were examined using chloroplast DNA (cpDNA) and microsatellite genotyping analyses for clarifying its invasion route and process. According to the cpDNA analysis, *S. alterniflora* populations in Japan had a single haplotype (haplotype C4) that is the most dominant genotype around the Florida Peninsula, the region of its origin, and is also widely found in the introduced populations in the East Asia. Microsatellite analysis also showed a loss of genetic diversity in Japanese *S. alterniflora* populations (allelic richness (*A*_R_) = 1.20–1.39) compared with that in its native region (*A*_R_ = 4.58–4.59), suggesting a founder effect on *S. alterniflora* that might have occurred after invasion of the species into Japan. The principal coordinate analysis and The STRUCTURE analysis indicated that no gene mixing among Japanese local populations (Aichi, northern and southern Kumamoto) was observed, indicating that *Spartina* invasion occurred independently into these regions. Among the three regions, trading between the ports of northern Kumamoto and the U.S. was obviously lower than trading with China. We concluded that invasive *S. alterniflora* might have independently invaded Japan at different times through an East Asia route, particularly *via* China (i.e., secondary introduction). Therefore, it is important to strengthen the quarantine control on the importation of commodities, especially of transport vehicles at potential donor spots (i.e., border control/border biosecurity system), and to share information networks on invasive species between each region/port for minimizing further risks of biological species such as *Spartina*

## Introduction

Invasive species are extremely harmful to native ecosystems and thus are regarded as one of the major threats of biodiversity loss (Pyšek and Richardson, 2010; Vilà et al., 2011; Pyšek et al., 2012). International trade serves as one of the driving factors for the widespread invasion of the invasive species (Elton, 1958; Lockwood et al., 2007; Davis, 2009; Richardson, 2011). Many empirical and theoretical studies on biological invasions have been conducted on various taxonomic groups for resolving this worldwide concern (Lee, 2002). Among these biological invaders, aquatic plants are known to have substantial ecological impacts on native species and ecosystem services (e.g., Hayasaka et al., 2018), as well as subsequent huge economic losses. Therefore, ecological knowledge that may lead to urgent control and/or eradication of invasive aquatic plants are imperative to conserve a biological diversity (Koncki and Aronson, 2015).

Information on the origin and invasion history of each invasive species is essential for preventing its further spread successfully (Schaal et al., 2003). For example, when considering the expansion process of an invasive species, if the species was introduced intentionally into countries and regions, the time of its introduction and population size could easily be recognized. In contrast, it is very difficult to obtain such information on biological invaders when due to unintentional introductions. The invasion history of invasive species, especially plants, are estimated directly, for example, using published literature, aerial photographs, and herbarium collections in order to determine the date and place of its first record. On the other hand, molecular genetic data including population genetic structure and diversity can provide a great deal of information, such as the origin of the targeted species and the route of its propagation, as well as the process of the range expansion, which indirectly contributes to the elucidation of its invasion history (Lowe et al., 2004; Prentis et al., 2009; Hoos et al., 2010; Lombaert et al., 2010). Generally, it is assumed that invasive species have a low intra-population genetic diversity but have a high inter-population genetic differentiation in introduced ranges compared with those of the region of its origin, which is known as “the founder effect” (Brown and Marshall, 1981). However, the degree of genetic diversity and differentiation of introduced populations obviously varies for each invasion event (e.g., Amsellem et al., 2000; McCauley et al., 2003; Provan et al., 2005). For example, Bossdorf et al. (2005) indicated that multiple introductions of invasive populations appear to be the rule rather than the exception, while other researchers have reported that the frequency of introductions may greatly contribute to the decrease of genetic diversity in these populations if a highly competitive species has invaded a region rich in genetic diversity, and to the relief from inbreeding depression over the short run (years to decades) (e.g., Frankham et al., 2002; Saltonstall, 2002; Dlugosch and Parker, 2008). In addition, the genetic characterization of a population is largely associated with the ability of distribution expansion (Lee, 2002). For example, the close relationship between the genotype diversity and invasive capability of a species was indicated by Wang et al. (2012). Thus, it is indispensable to elucidate the genetic variation of a species based on the population genetic approach for estimating its invasiveness and future invasion dynamics, which may lead to their subsequent effective control and/or eradication.

In Japan, *Spartina alterniflora* Loisel (smooth cordgrass), a plant native to the Atlantic coast of North America and the Gulf of Mexico, was first detected in 2008 in Aichi Prefecture and in 2009 in Kumamoto Prefecture, followed by identification in multiple rivers and tidal flats in both prefectures (i.e., unintentional introduction) (Tamaoki and Takizaki, 2015). However, the reason why *S. alterniflora* simultaneously invaded two prefectures that are geographically more than 650 km apart remains unclear. The *Spartina* spp. (cordgrass) (Bortolus et al., 2019) greatly alter brackish and estuarine salt marsh environments *via* changes in the sediment properties of the tidal flats with growth, resulting in its subsequent further population expansion (e.g., Howes and Teal, 1994; Neira et al., 2005). In addition, serious ecological impacts of *Spartina* species on native aquatic ecosystems through competitive exclusion (Goss-Custard and Moser, 1988; Wan et al., 2009; Zhou et al., 2009; Morgan and Systma, 2010) and changes in community and trophic structures (Simenstad and Thom, 1995; Levin et al., 2006; Bortolus et al., 2015) were found due to their expansion. Accordingly, *Spartina anglica* C.E. Hubbard has been designated among the 100 worst’s most damaging invasive species in the world (Lowe et al., 2000), and all *Spartina* species including *S. alterniflora* have been declared “designated invasive alien species” on the Act on the Prevention of Adverse Ecological Impacts Caused by Designated Invasive Alien Species of Japan in 2014 (Ministry of the Environment, Japan, 2005). To achieve control and/or eradication of invasive *S. alterniflora* and prevent its future invasion successfully, knowledge about the current status of *S. alterniflora* in Japan through a population genetic approach is thought indispensable.

There are some studies that compared the genetic variation of *S. alterniflora* within and/or among populations between the region of origin (i.e. the Atlantic coast of the United States) (Blum et al., 2007) and the introduced (China) or invaded (i.e., the Pacific coast of the U.S. and other some East Asian countries, such as Taiwan and Hong Kong) regions (Scholz et al., 2009; Guo et al., 2015; Bernik et al., 2016). For example, the most likely invasion pathways of *S. alterniflora* in Willapa Bay, Washington, on the Pacific coast of the U.S. was the transport and translocation of oysters for cultivation *via* interstate railroad after the 1890s (Civille et al., 2005). This fact suggests that *S. alterniflora* populations in the Willapa Bay might be derived from multiple populations on the Atlantic coast around New York (i.e., mid-Atlantic source) (Blum et al., 2007). Also, Blum et al. (2007) estimated that *S. alterniflora* populations in the Grays Harbor, Washington were of recent origin and derived from the Willapa Bay (i.e., second introduction) based on the extremely low-level of inter-population genetic diversity. On the other hand, populations of this species in the San Francisco Bay, California, and China, which were introduced intentionally, had a relatively high genetic diversity (Blum et al., 2007; Bernik et al., 2016).

Here, the genetic structure of invasive *S. alterniflora* in Japan and its origin were assessed by analyzing the degree of genetic diversity and genetic mixing in Japanese populations, using chloroplast and nuclear molecular markers. Then, the genetic variance of *S. alterniflora* was compared between populations in the region of origin (the eastern U.S.) and those in several introduced regions (the Pacific coast of the U.S. and some East Asian countries). This study will elucidate whether actual invasion route of *S. alterniflora* into Japan was derived from the region of origin (i.e., primary introduction) or from a secondary introduction *via* introduced regions. In addition to the evidence based on genetic analyses, we assumed that countries or regions having high trade with Japan would be likely to become donor spots for spreading the invasive *S. alterniflora* irrespective of intentional/unintentional pathways. Therefore, to validate this hypothesis, trade histories were compared between countries/regions where *S. alterniflora* has grown naturally (the United States, the East Asian countries) and Japan (Aichi and Kumamoto Prefectures). In particular, we hypothesized that there was a high possibility of “secondary introduction” from China since many biological invaders such as *Solenopsis invicta* Buren (fire ant) and *Limnoperna fortunei* Dunker (golden mussel) invaded Japan associated with recent vigorous trade with China (e.g., Magara et al., 2001; Murakami, 2018).

## Materials and Methods

### Sampling Collection and DNA Extraction

**Figure 1:**
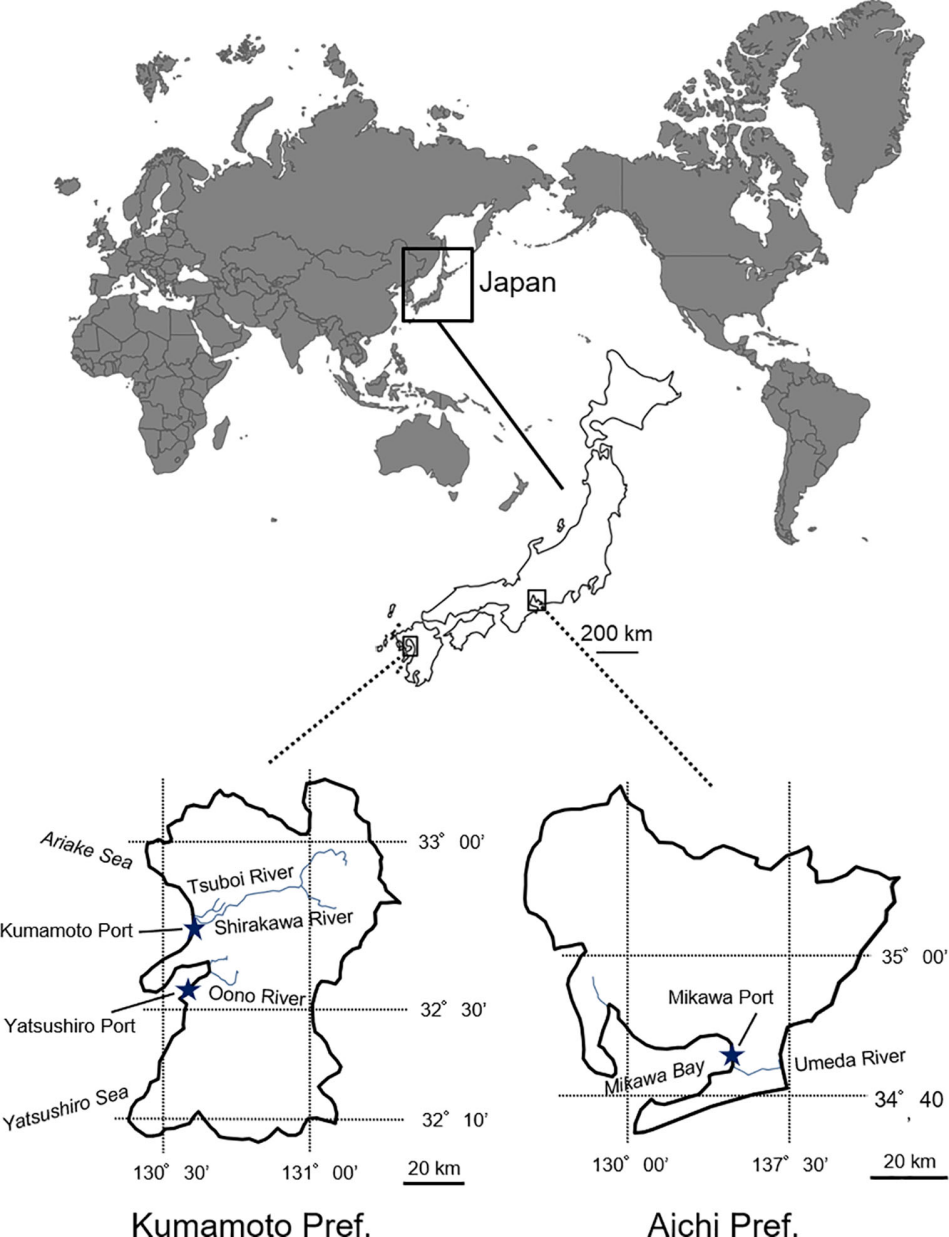
Invasion areas (Aichi and Kumamoto Prefectures) of invasive *Spartina alterniflora* in Japan.

*Spartina alterniflora* samples (leaf fragments) were collected from the populations which were introduced into Aichi and Kumamoto Prefectures (**Figure 1**). In Aichi Prefecture, 27 samples (i.e., one individual per colony) were collected from multiple colonies along and around the Umeda River (N34° 42′, E 137° 18′) facing the Mikawa Bay. In Kumamoto Prefecture, 20 and 19 *S. alterniflora* samples were randomly collected from multiple colonies in the Tsuboi River (N 32° 46′, E 130° 37′) facing the Ariake Sea (northern Kumamoto) and the Oono River (N32° 37′, E 130° 39′) facing the Yatsushiro Sea (southern Kumamoto), respectively. In contrast, only three samples from three colonies were collected in the estuary of the Shirakawa River (N 32° 46′, E 130° 36′) facing the Ariake Sea (northern Kumamoto) because almost all the populations had been eradicated by 2012 to 2015 by drawing out and backhoe dredger (**Figure 1**). The sample collection was carried out following the method in Blum et al. (2007), who indicated that samples should be collected from colonies that are at least about 2.5 m apart from each other (**Supplementary Table 1**). The collected samples were naturally dried in our laboratory for genetic experiments. Total DNA was extracted from a 0.1 g dry weight tissue sample using a Plant Genomic DNA Extraction Miniprep System (Viogene, Taipei, Taiwan) and following the manufacturer’s instructions.

### Chloroplast DNA Sequencing and Data Analysis

To compare the chloroplast DNA (cpDNA) haplotypes of *S. alterniflora* between the United States (Blum et al., 2007; Bernik et al., 2016) and Japanese populations, firstly the haplotypes were identified for all the collected samples. For polymerase chain reaction (PCR) amplification and sequencing of the *trnT*–*trnF* region of cpDNA, two primer pairs were used: Tab A (5′-CAT TAC AAA TGC GAT GCT CT-3′) and Tab B (5′-TCT ACC GAT TTC GCC ATA TC-3′) targeting the *trnT*–*trnL* region; and Tab C (5′-CGA AAT CGG TAG ACG CTA CG-3′) and Tab F (5′-ATT TGA ACT GGT GAC ACG AG-3′) targeting the *trnL*–*trnF* region were used (Taberlet et al., 1991). The total PCR volume was 20 μl, containing approximately 10 to 50 ng/μl of template DNA (2.0 μl), 10× NH_4_ reaction Buffer (2.0 μl), 10 mM dNTP mix (1.6 μl), 50 mM MgCl_2_ (1.6 μl), 0.2 μl of each 100 pM primer pair, and 5 U/µl of Biotaq™ DNA polymerase (0.1 μl) (Nippon Genetics, Tokyo, Japan) were used. The temperature conditions of Blum et al. (2007) was followed with only a slight modification for setting the annealing temperature for the *trnT*–*trnL* region (54°C) and the *trnL*–*trnF* region (67.5°C). A TaKaRa PCR Thermal Cycler (TaKaRa BIO, Shiga, Japan) was used for the PCR assay. PCR products were purified using NucleoSpin Extract II (Macherey–Nagel, Düren, Germany) and then were used as a template for the cycle sequencing reaction. The cycle sequencing reaction assay was conducted by Macrogen Japan Corporation (Kyoto, Japan) and analyzed using a 3730*xl* DNA analyzer (Applied Biosystems, Foster City, CA). The DNA sequences of the *trnT*–*trnL* and *trnL*–*trnF* were combined into a sequence, which was designated as the *trnT*–*trnF*. The base sequence of the *trnT*–*trnL* obtained in this study was compared with the existing 42 haplotypes in *S. alterniflora* (accession numbers AY927278–AY927299 and DQ486839–DQ486858) (Blum et al., 2007) in order to determine its haplotype. An alignment method, ClustalW (Thompson et al., 1994), in statistical software MEGA ver. 6.0 was used for competitive multiple sequence alignment (MSA) (Tamura et al., 2013). All names of the haplotypes obtained in this study were assigned according to the method of Blum et al. (2007).

### Microsatellite Analysis and Data Analysis

Microsatellite analysis was conducted using 11 microsatellite markers (SPR1, SPR2, SPR3, SPR4, SPR5, SPR6, SPR7, SPR8, SPR9, SPR10, SPR11), developed by Blum et al. (2004). The PCR amplification were carried out in a total volume of 20 μl, consisting of approximately 10 to 50 ng/μl template DNA (4.0 μl), 10× Buffer (2.0 μl), 2 mM dNTP mixture (2.0 μl), 0.2 μl of each 100 pM primer pair, and 2.5 U/μl of Blend Taq (0.5 μl) (TOYOBO, Osaka, Japan). The forward primer was fluorescently labeled with 5′-FAM, TAMRA, and 5′-JOE. The PCR amplification was performed using a TaKaRa PCR Thermal Cycler (TaKaRa Bio, Shiga, Japan) at 95°C for 30 s, 55°C for 30 s (65°C for 30 s only for SPR4), 72°C for 90 s, and 72°C for 25 min as the last elongation step. Fragment analysis was conducted by Macrogen (Seoul, South Korea). Based on the microsatellite analysis, individuals which had an exact genotype match were considered as “clones” and then were excluded from the analyses. The genotypes of *S. alterniflora* populations in Japan were identified using 11 different microsatellite markers (**Supplementary Table 2**). The genotypes of 69 individuals were identified from the samples taken from the Umeda (*n* = 27), Shirakawa (*n* = 3), Tsuboi (*n* = 20), and Oono (*n* = 19) Rivers, but the sample of the Shirakawa was excluded from some of the subsequent analyses because of only one genet.

To compare the degree of genetic diversity of *S. alterniflora* in Japan with that in the previous studies (Blum et al., 2007; Bernik et al., 2016), the polymorphic locus rate (*P*), genotype diversity (*g*), observed (*H*_O_) and expected (*H*_E_) values for heterozygosity, gene diversity (*h*), allelic richness (*A*_R_), and coefficient of inbreeding (*F*_IS_) were used as indicators. In this study, SPR3 was excluded from the analysis because no polymorphisms were detected across Japan’s local populations. The polymorphic locus rate (*P*) was calculated for each local population. This value indicates the rate of genetic loci with polymorphisms compared to all the genetic loci for each local population. Presence/absence of the multilocus genotype matches in among individual polymorphic gene loci was analyzed using Software GENALEX ver. 6.5 (Peakall and Smouse, 2012). The genotype diversity (*g*) for each *S. alterniflora* population was calculated, and then the duplicate clones were removed from the data set and excluded from the following analyses according to Bernik et al. (2016). The value of *g* indicates the rate of the individuals with duplicate clones removed in each local population. The observed (*H*_O_) and expected (*H*_E_) values for heterozygosity were calculated using GenAlEx ver. 6.5 (Peakall and Smouse, 2012). The gene diversity (*h*), allelic richness (*A*_R_), and coefficient of inbreeding (*F*_IS_), and its confidence intervals were calculated using FSTAT ver. 2.9.3 (Goudet, 2001). Tests for deviation from Hardy–Weinberg equilibrium (HWE) were also performed using FSTAT ver. 2.9.3 (Goudet, 2001).

To verify whether a genetic bottleneck had been formed in each local population in Japan, BOTTLENECK analysis (Piry et al., 1999) was conducted using Wilcoxon’s heterozygosity excess test (Luikart et al., 1998a) and the mode shift test (Luikart et al., 1998b). This is because Piry et al. (1999) suggested that Wilcoxon’s test is most powerful and robust when used with few polymorphic loci. Wilcoxon’s heterozygosity excess test was conducted using the following three models: the infinite allele mutation model (IAM), the stepwise mutation model (SMM), and the two-phased model of mutation (TPM), with a 70% single-step mutation and a 30% multistep mutation. Regarding the genetic differences among the individuals, the pairwise co-dominant genotypic distances in each Japanese population were calculated using GenAlEx ver. 6.5 and then evaluated by principal coordinate analysis (PCoA) (Peakall and Smouse, 2012).

To evaluate the genetic structure in the individuals, analysis with STRUCTURE allocated all individuals to K clusters by Bayesian’s clustering and was conducted to maximize the linkage disequilibrium and Hardy-Weinberg’s disequilibrium. Software STRUCTURE ver. 2.3.4 (Pritchard et al., 2000) was used for this analysis. Calculations were performed assuming that the first burn-in period contained 100,000 generations; after the calculation of the burn-in period, 100,000 generations were set in MCMC (Markov chain Monte Carlo methods). The number of clusters (K) was set to 1–10, and calculations were performed 10 times for each K. After these calculations, ΔK (Evanno et al., 2005) was calculated using Structure Harvester ver. 0.6.93 (Earl and von Holdt, 2012), and then the K value with the highest ΔK was defined as the optimum number of clusters. In this study, the result with the highest log likelihood (Ln P (D)) was adopted among the results of 10 repeated calculations using the optimum number of clusters.

## Results

### Chloroplast DNA Haplotype Diversity of *Spartina alterniflora*

**Figure 2:**
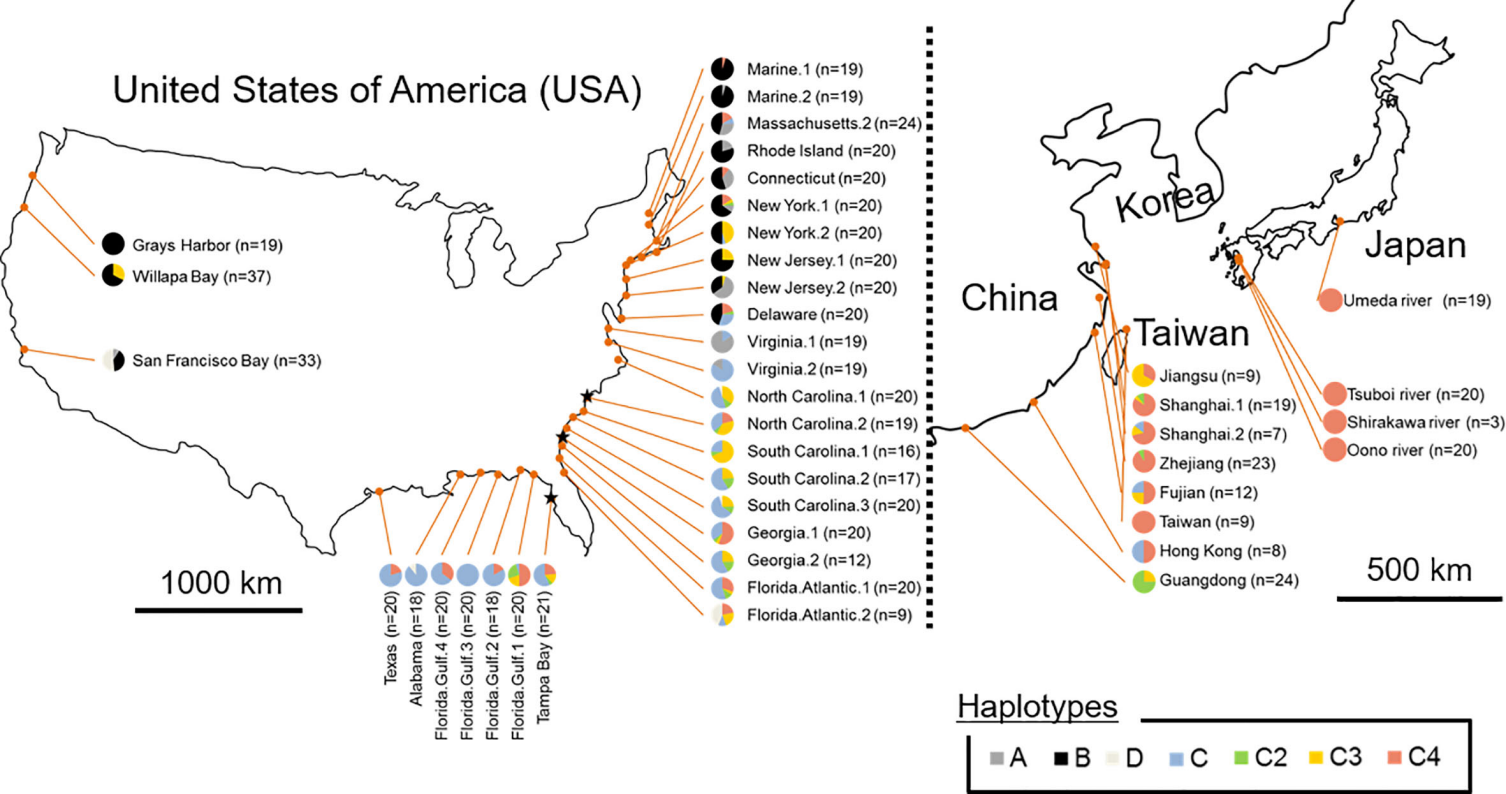
Frequency and distribution of chloroplast DNA (cpDNA) haplotypes in the region of origin (the eastern Unites States) and in the regions where *Spartina alterniflora* had been introduced intentionally and/or unintentionally (the Pacific coast of the U.S. and the East Asian countries). Groups A, B, and D consisting of a single haplotype are shown in dark grey, black and light grey, respectively. Haplotype C2, C3, and C4 of Group C consisting of multiple haplotypes are shown in green, yellow, and pink, respectively, and other C members are shown in blue. ★ indicates the region estimated as the place that *S. alterniflora* was initially introduced into China, according to Bernik et al. (2016).

The sequences of *trnT*–*trnF* region from chloroplast DNA were identified from all *S. alterniflora* individuals sampled in both prefectures and regions: the Umeda River (Aichi), the Shirakawa River and Tsuboi River (northern Kumamoto), and Oono River (southern Kumamoto). The sequences of *trnT*–*trnF* region from Japanese populations revealed that all *S. alterniflora* populations in Japan had a single haplotype (accession number: LC565815): the haplotype C4 (accession number: KJ499448, Guo et al., 2015; MG201950, Qiao et al., 2019) (**Figure 2**, **Table 1**). Haplotype C4 has been identified as widespread in the Atlantic coast of the U.S., especially around the Florida Peninsula. Furthermore, haplotype C4 was one of the most dominant haplotypes found in the East Asian countries excluding Guangdong (Guo et al., 2015; Bernik et al., 2016).

**Table 1:** Information on the genetic diversity of invasive *Spartina alterniflora* based on the microsatellite loci in Japan.

Regions	*N*_site_	*N*_μsat_	*g*	*P*	*H*_O_	*H*_E_	*h*	*A*_R_	*F*_IS_	Data reference
Native range	25	400	0.93 _(0.12)_	0.88 _(0.11)_	0.44 _(0.12)_	0.56 _(0.08)_	0.42 _(0.08)_	4.59 _(1.24)_	0.13 _(0.20)_	Blum et al., 2004 Bernik et al., 2016
*North and Mid-Atlantic* (Marine, Massachusetts, Rhode Island, Connecticut, New York, New Jersey, Delaware, Virginia, North Carolina)	15	223	0.98 _(0.05)_	0.89 _(0.11)_	0.37 _(0.09)_	0.54 _(0.08)_	0.43 _(0.09)_	4.59 _(1.41)_	0.22 _(0.13)_	
*Southeastern U.S.* (North Carolina, South Carolina, Georgia, Florida Atlantic, Tampa Bay, Florida Gulf)	10	177	0.87 _(0.17)_	0.86 _(0.11)_	0.55 _(0.08)_	0.59 _(0.06)_	0.41 _(0.06)_	4.58 _(0.98)_	−0.02 _(0.17)_	
Introduced or invasion ranges	8									
*Pacific Coast of the U.S.* (Willapa Bay)	1	21	0.95	1.00	0.33 _(0.31)_	0.46 _(0.28)_	0.44 _(0.25)_	4.25 _(2.61)_	0.11	Blum et al., 2004 Bernik et al., 2016
*China*	3	67	0.77 _(0.39)_	0.88 _(0.00)_	0.55 _(0.13)_	0.53 _(0.06)_	0.47 _(0.05)_	3.52 _(0.46)_	−0.02 _(0.16)_	Bernik et al., 2016
Shanghai	(1)	19	1.00	0.88	0.49 _(0.27)_	0.52 _(0.21)_	–	3.71 _(1.38)_	0.04	
Zhejiang	(1)	23	1.00	0.88	0.45 _(0.20)_	0.51 _(0.23)_	–	3.86 _(1.95)_	0.10	
Guangdong	(1)	25	0.32	0.88	0.70 _(0.20)_	0.61 _(0.14)_	–	3.00 _(1.16)_	−0.21	
*Japan*	4	69								This study
Aichi Prefecture	(1)									
Umeda River		27	0.89	0.70	0.33 _(0.09)_	0.33 _(0.07)_	0.34	1.34 _(0.22)_	0.01	
Kumamoto Prefecture										
Northern Kumamoto	(2)									
Shirakawa River		3	0.33	0.00	0.20 _(0.13)_	0.10 _(0.07)_	–	1.20 _(0.40)_	–	
Tsuboi River		20	0.80	0.60	0.18 _(0.06)_	0.23 _(0.08)_	0.24	1.24 _(0.24)_	*0.29	
Southern Kumamoto	(1)									
Oono River		19	1.00	0.80	0.30 _(0.08)_	0.38 _(0.06)_	0.39	1.39 _(0.20)_	*0.24	

N_site_ shows the number of samples of S. alterniflora in each region. N_µsat_ indicates the number of individuals for microsatellite analysis in each region. The indicators related to the genetic analysis are as follows: g, genotype diversity; P, polymorphic locus rate; H_O_, observed value for heterozygosity; H_E_, expected value for heterozygosity; h, gene diversity; A_R_, allelic richness; F_IS_, coefficient of inbreeding. Numbers of parenthesis in “N_site_ column” represent numbers of breakdown samples in each region and those in other columns indicate the standard deviation of each indicator. *P < 0.05.

### Genetic Diversity of *Spartina alterniflora* Based on the Microsatellite Loci

Results of the genetic analysis of Japanese *S. alterniflora* samples collected using the different markers demonstrated that the number of alleles of *S. alterniflora* individual stands in each river was less than or equal to 2, except for one sample from the Tsuboi River (**Supplementary Table 2**). Among the 11 microsatellite markers, no genetic polymorphisms were detected from the locus SPR3. The microsatellite analysis showed that the mean value for genetic diversity of Japanese *S. alterniflora* samples were as follows; the Umeda River (*h* = 0.34, *A*_R_ = 1.34 ± 0.22), Tsuboi River (*h* = 0.24, *A*_R_ = 1.24 ± 0.24), and Oono River (*h* = 0.39, *A*_R_ = 1.39 ± 0.20).

The genotype diversity (*g*) was extremely high at 0.93 ± 0.12 in samples from the Atlantic coast of the U.S. (native range), and similar tendencies were also found in other regions where *S. alterniflora* invaded (**Table 1**). The *g* values of Japanese *S. alterniflora* populations, excluding the Shirakawa, lay within 0.80 to 1.00, almost equivalent to those in China (*g* = 0.77 ± 0.39) and the introduced in Willapa Bay (the Pacific coast of the U.S.) (*g* = 0.95). On the other hand, low *g* values were found in samples from the Shirakawa River (*g* = 0.33) and Guangdong province in China (*g* = 0.32), where almost all analyzed samples had the same genotype. The inbreeding coefficient (*F*_IS_) of each population in Japan indicated that estimated *F*_IS_ values of samples from the Tsuboi (*F*_IS_ = 0.29) and Oono (*F*_IS_ = 0.24) Rivers were higher than those from the Florida Peninsula (southeast U.S.) (*F*_IS_ = −0.02 ± 0.17) and China (*F*_IS_ = −0.02 ± 0.16), suggesting the significantly excessive homozygosity (*P*<0.05). However, the *F*_IS_ values of samples from the Umeda River (*F*_IS_ = 0.01) did not deviate from the Hardy-Weinberg equilibrium (**Table 1**). The significant excessive homozygosity on Japanese *S. alterniflora* populations was observed in the infinite allele mutation model (IAM), the stepwise mutation model (SMM), and the two-phased model of mutation (TPM) (*P*<0.05). In addition, the mode shift test suggested that a bottleneck (i.e., shifted mode) would have been formed in these populations in recent years (**Table 2**).

**Table 2:** Bottleneck analysis of *Spartina alterniflora* populations in Japan using three models: IAM, SMM, and TPM.

Samples(Population)	Region	Wilcoxon sign–rank test			Mode shift
		IAM	TPV	SMM	
Umeda River	Aichi Pref.	*	*	*	Yes
Oono River	Southern Kumamoto Pref.	*	*	*	Yes
Tsuboi River	Northern Kumamoto Pref.	*	*	*	Yes
Shirakawa River	Northern Kumamoto Pref.				

“yes” indicates the existence of a mode shift in allele frequency. The sample in the Shirakawa River was excluded from the analysis because all the samples collected in the Shirakawa River were the same genet. *P < 0.05.

### Genetic Distance Among *Spartina alterniflora* Local Populations in Japan

**Figure 3:**
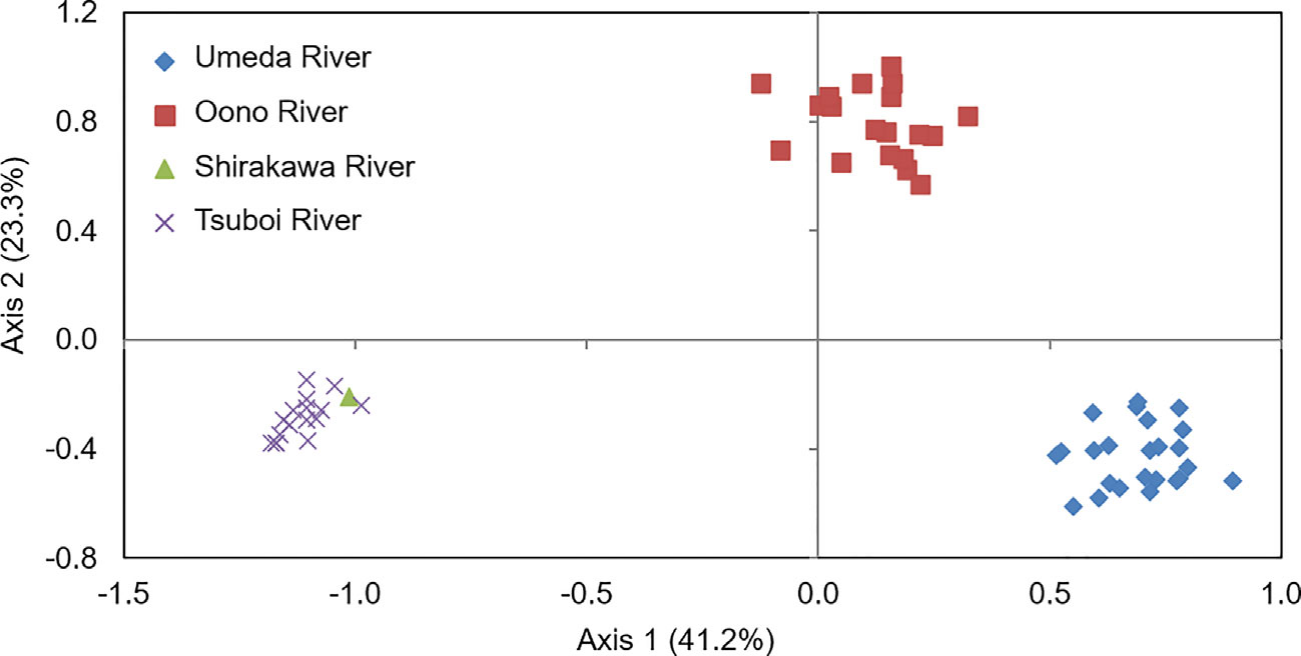
Results of a principal coordinate analysis (PCoA) of *Spartina alterniflora* local populations in Japan based on co-dominant genotypic distances. Axis 1 and Axis 2 account for 41.2% and 23.3% of the variance, respectively. Symbols are as follows: rhomboid, populations in Umeda River (Aichi); square, in Oono River (southern Kumamoto); triangle, in Shirakawa River (northern Kumamoto); cross, in Tsuboi River (northern Kumamoto).

Principal coordinate analysis (PCoA) based on co-dominant genotypic distances revealed that genetic distances of *S. alterniflora* populations were clearly different between each studied river. The proportions for Axis 1 and Axis 2 were 41.2% and 23.3%, respectively. Although *S. alterniflora* populations in the Shirakawa and Tsuboi Rivers were placed in the same position, those in the Oono and Umeda Rivers were clearly separated along Axis 1 (**Figure 3**), suggesting that there were at least three *S. alterniflora* local populations in Japan.

**Figure 4:**
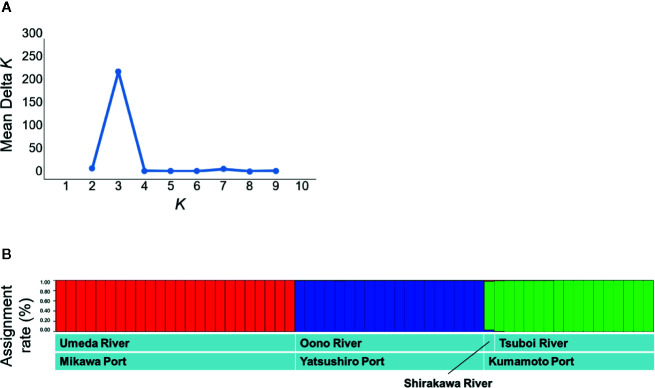
Population structures based on the microsatellite mutation among the genes sampled from *Spartina alterniflora* populations in Japan using Bayesian estimation. **(A)** The estimation of the optimum number of clusters based on ΔK. **(B)** The assignment of each individual into the clusters using STRUCTURE analysis. The vertical axis in panel B shows attributed rates of each sample collected in *S. alterniflora* local populations.

The STRUCTURE analysis indicated that the studied populations were divided into distinct genetic clusters. The ΔK value was clearly the highest at K = 3 (**Figure 4A**). As a result, *S. alterniflora* populations of Japan were classified into three groups: 1) Umeda River (Aichi), 2) Shirakawa and Tsuboi Rivers (northern Kumamoto), and 3) Oono River (southern Kumamoto) (**Figure 4B**). In addition, each group was practically unmixed with any other group.

## Discussion

### Invasion Process and Route of Invasive *Spartina alterniflora* in Japan

Genetic analysis of cpDNA revealed that all *S. alterniflora* populations in Japan had a single haplotype (haplotype C4) (**Figure 2**, **Table 1**). Within the region of its origin, haplotype C4 was widely observed in the Atlantic coast of the U.S. Also, this haplotype was the most dominant in the East Asian countries where *S. alterniflora* has been introduced intentionally (China, see An et al., 2007) or unintentionally (Taiwan and Hong Kong e.g., Scholz et al., 2009; Guo et al., 2015). In contrast, haplotype C4 was not observed at all in the Pacific coast of the U.S. (Blum et al., 2007; Guo et al., 2015; Bernik et al., 2016). Therefore, this finding suggests that *S. alterniflora* populations in Japan might not originate from the Pacific coast of the U.S. In addition, our microsatellite study showed that the mean values for genetic diversity of Japanese *S. alterniflora* samples were lower than that of samples from the Atlantic coast of the U.S. (*h* = 0.42 ± 0.08, *A*_R_ = 4.59 ± 1.24) and the Florida Peninsula (southeast U.S.) (*h* = 0.41 ± 0.06, *A*_R_ = 4.58 ± 0.98), the region of its origin (Blum et al., 2007; Bernik et al., 2016), and China (*h* = 0.47 ± 0.05, *A*_R_ = 3.52 ± 0.46) (Bernik et al., 2016) and Willapa Bay (*h* = 0.44 ± 0.25, *A*_R_ = 4.25 ± 2.61) located in the Pacific coast of the U.S. (Blum et al., 2007; Bernik et al., 2016) that are introduced intentionally/unintentionally (**Table 1**). It should be noted that no information has been reported on *S. alterniflora* populations with such low genetic diversity so far (Blum et al., 2007; Bernik et al., 2016). Indeed, values of *A*_R_ in Japanese *S. alterniflora* were very low in comparison to those in the U.S. and China populations, with significant excessive homozygosity detected in three mutation models. In addition, the formation of a bottleneck (i.e., shifted mode) was expected by the mode shift test in *S. alterniflora* population in Japan. Therefore, these results reveal that the founder effect might have occurred in Japanese *S. alterniflora* population. Such low genetic diversities associated with a founder effect were also found in other *Spartina* species such as *S. versicolor* Fabre introduced in Europe (Baumel et al., 2016) and *S. densiflora* Brongn. invading the Pacific coast of the U.S. (Castillo et al., 2018). Since the cause of a lower genetic diversity among invasive *Spartina* species is of great interest, we discuss below the reason why *S. alterniflora* populations had lower genetic diversity when invading Japan.

After 1979, seeds and individuals of *S. alterniflora* were intentionally introduced into China from multiple areas of the Atlantic coast of the U.S. (e.g., North Carolina, Georgia, and Florida) for eco-engineering purposes (i.e., reclamation of tideland) (Xu and Zhou, 1985; Wan et al., 2009). The coverage of *S. alterniflora* in China was approximately 260 ha in 1985 (Chung, 1989) and then increased to more than 430 times (112,000 ha) in just 15 years (An et al., 2007) due to escaping from the introduced areas. These facts suggest that *S. alterniflora* expanding in East Asian countries originates from populations (found) in the southeast U.S., especially around the Florida Peninsula. Populations of *S. alterniflora* in the Grays Harbor, Washington (haplotype B) and Taiwan (haplotype C4), which had only a single haplotype as well as Japan (**Figure 2**), were unintentionally and secondarily introduced from the Willapa Bay, Washington (the Pacific coast of the U.S.) (Civille et al., 2005) and the vicinity of Fujian (China) (Lin et al., 2015), respectively.

In this study, we assumed that countries or regions having high trade with Japan would be likely to become donor spots for spreading the invasive *S. alterniflora* irrespective of intentional/unintentional pathways as mentioned before. Undoubtedly, this can be generalized regardless of taxonomic groups. Thus, to validate this hypothesis, trade histories were compared between countries/regions where *S. alterniflora* has grown (the United States, China, Taiwan, Hong Kong) (Blum et al., 2007; Guo et al., 2015; Bernik et al., 2016) and the ports nearest to each studied river in Japan (i.e., Kumamoto Port, Yatsushiro Port, and Mikawa Port) using historical trade data from the 2003 to 2013 in the Global Trade Atlas (https://www.gtis.com/gta/). The findings revealed that when compared the amount of trade between the Yatsushiro Port (southern Kumamoto), which includes the Oono River and the U.S. ($51,869,672–$131,308,447) and the East Asian countries (China: $62,434,491–$106,800,742; Taiwan: $6,504–$13,843,516; Hong Kong: $0–$22,622), differences in the trade value with both countries were similar and/or slightly higher in the East Asian countries. Similar trend on the amount of trade with U.S. ($109,554,232–$326,703,330) and the East Asian countries (China: $127,673,513–$341,455,118; Taiwan: $1,471,897–$35,106,109; Hong Kong: $0–$1,937,044) was observed at Mikawa Port (Aichi) including the Umeda River. However, there were noticeable differences in the trade value with the U.S. ($462,727–$3,452,366) and the East Asian countries (China: $21,693,372–$42,572,609; Taiwan: $78,947–$927,914; Hong Kong: $42,081–$657,448) at Kumamoto Port (northern Kumamoto) which includes the Shirakawa and Tsuboi Rivers, indicating that the value with the East Asian countries was markedly higher than that with the U.S. These data suggest that the route through which invasive *S. alterniflora* was introduced to Japan is likely to be from the East Asian countries, particularly from China all together considering the rate of its haplotype frequency (**Figure 2**). The East Asian countries are one of the largest supply sources on young shellfish and seedlings for cultivation in tidal flats of Japan (Okoshi, 2007), and thus the contamination of multiple species of organisms is often observed with the imports. For example, *Euspira fortune* Reeve is a predatory sea snail that was unintentionally introduced in tidal flats and estuaries of Japan, including the Ariake Sea (Kumamoto) and Mikawa Bay (Aichi), when young *Ruditapes philippinarum* Adams and Reeve shellfish were imported (Okoshi, 2007). Similarly, *S. alterniflora* in the western U.S. was also introduced unintentionally from the eastern U.S. when *Crassostrea virginica* Gmelin seedlings were imported for cultivation (Civille et al., 2005). From these facts, we cannot deny the possibility that *S. alterniflora* was introduced unintentionally into Japan through the importation of cultured shellfishes. Therefore, further research on the genetic characteristics of the invasive *S. alterniflora* should be carried out worldwide for estimating its global spread and future invasion risks.

### Genetic Relationships Between Japanese *Spartina alterniflora* Local Populations

Comparison of microsatellite data among *S. alterniflora* local populations in Japan for estimating the route through which *S. alterniflora* invaded Japan revealed that genotypes of the populations were clearly different in each river (**Figures 3** and **4**). These results suggest that there is no exchange of *S. alterniflora* genome among the four rivers in Japan. Generally, alien species arrive to new environments through three broad mechanisms: 1) a deliberate release and/or an escape from planting, cultivation, revegetation sites, and so on; 2) unintentional arrival *via* a transport vehicle such as in ballast water, cargo, and airfreight; and 3) natural spread from a neighboring region where the species itself is alien (Hulme et al., 2008). Among these invasive mechanisms, the possibility of *S. alterniflora* invasion in Japan *via* intentional introductions is almost impossible, since Japan has no such imports for the reclamation of tidal flats. Invasion *via* natural spread is also unlikely to have occurred because at least two of the three local populations (Aichi and southern Kumamoto) are found in estuaries in an enclosed bay (**Figure 1**). Therefore, the most likely invasion route may have been the arrival through a transport vehicle (i.e., stowaway) (Hulme et al., 2008).

Results of the microsatellite analysis made it clear that some *S. alterniflora* individuals (St. 13, 15, 16, and 18) in the Tsuboi River (northern Kumamoto) had a heterozygous at only one locus, while two individuals growing sympatrically (St. 14 and 17) had a homozygous at all of the loci (**Supplementary Table 2**). *Spartina alterniflora* rapidly spread their populations *via* both sexual (seed) (Hayasaka et al., 2020) and asexual (clonal) reproduction and then form a high-density single colony (Somers and Grant, 1981; Davis et al., 2004). It is suggested that although these individuals have actually grown *via* seed propagation (i.e., sexual reproduction), they may be considered as clones with exactly the same genotype due to the extreme homozygosity. The number of alleles per marker on each *S. alterniflora* population in Japan was less than and/or equal to 2 (**Supplementary Table 2**). Again, values of *A*_R_ in *S. alterniflora* populations in Japan were lower than those in the U.S. and China populations, and the formation of a bottleneck was expected in Japanese populations. Therefore, these facts indicate that the founder effect might have occurred in *S. alterniflora* populations in Japan. In addition, no *S. alterniflora* population was found in Japan before 2008 (Tamaoki and Takizaki, 2015). In other words, only a few individuals of *S. alterniflora* might have successfully invaded Japan. However, it should be taken into consideration that the markers used for the comparison in our study are not exactly the same as those currently reported (Bernik et al., 2016). Nevertheless, it suggests that only one *S. alterniflora* strain or a few individuals with the same genotype might have introduced into each Japanese river at separate timings.

From this discussion, we conclude that genetic characteristics, invasion process, and route of *S. alterniflora* populations in Japan were as follows: 1) all *S. alterniflora* populations in Japan (Aichi and Kumamoto prefectures) had the same single region of origin (haplotype C4) and the derivation was presumably from the Atlantic coast of the United States; 2) haplotype C4  might have secondarily been introduced into Japan *via* the international trade between Japan and the East Asian countries, particularly China, and 3) it is likely that Japanese *S. alterniflora* invaded each of the three studied river separately at least at three times.

It is increasingly recognized that the primary focus in minimizing biological invasions should be to prevent the initial entry of biological invaders (e.g., Williams and West, 2000; Saccaggi et al., 2016). Therefore, it is important to strengthen the quarantine control on the importation of commodities, especially of transport vehicles at potential donor spots (i.e., border control/border biosecurity system), to decrease further risks of various biological invaders (Chornesky and Randall, 2003; Xu et al., 2006) including that of *Spartina* species (Castillo et al., 2018; Gallego-Tévar et al., 2019). Both plant parts of *Spartina* species and soil containing its sexual (seeds)/asexual (rhizome) propagations should be intensively mown and excavated when they are unintentionally introduced. Simultaneously, it is also important that *S. alterniflora* is detected and eliminated at an early invasion stage in order to minimize its invasion. In this study, we predicted the low frequency of *S. alterniflora* invasion. The reason was that the number of alleles per marker on each *S. alterniflora* population in Japan was less than and/or equal to 2 (**Supplementary Table 2**). Despite this, it took approximately 6 years from its first detection to the start at the eradication project. As mentioned above, trade with China is extremely large. Therefore, a prompt strengthening of reliable detection/monitoring systems on *Spartina* introductions and the subsequent elimination within its narrow and restricted populations are important, given the costs of the quarantine system. For this purpose, it is essential to continue monitoring areas where *S. alterniflora* has already invaded. Furthermore, given that ports that trade with China are all over Japan, the strengthening of shared information networks on introduced species between each region/port would lead to minimize their biological invasion risks.

## Data Availability Statement

The datasets generated for this study can be found in the DNA Data Bank of JAPAN (DDJB), accession number: LC565815.

## Author Contributions

YM, MT, and DH designed and coordinated the research. YM, TH, AN, and DH collected samples. YM, MT, and YI analyzed the data. YM and DH drafted the paper with the input of NN. All authors contributed to the article and approved the submitted version.

## Funding

Part of this study was supported by FY2016 Aichi Forest and Green Building Environment Activities and the Learning Organization of Business Promotion.

## Conflict of Interest

The authors declare that the research was conducted in the absence of any commercial or financial relationships that could be construed as a potential conflict of interest.
