# Supplementary material for: Genetic Diversity of Invasive Spartina alterniflora Loisel. (Poaceae) Introduced Unintentionally Into Japan and Its Invasion Pathway
Source: Front Plant Sci. 2020 Sep 7;11:556039. doi: 10.3389/fpls.2020.556039 (PMC7503347; doi:10.3389/fpls.2020.556039)
Supplement: Supplementary file 1 [file Table_1.docx]

**Supplementary Table 1** Geographical distance among the four studied river (Umeda River (Aichi), Oono River (southern Kumamoto), Shirakawa, and Tsuboi Rivers (northern Kumamoto)) and the maximum / minimum ranges between *Spartina alterniflora* samples (i.e., colonies) collected in each river.

| **Geographical distance between each studied river**  **(km)** | **Umeda River** | **Oono River** | **Shirakawa River** | **Tsuboi River** |  | **Range between *Spartina alterniflora* samples collected in each studied river**  **(m)** | |
| --- | --- | --- | --- | --- | --- | --- | --- |
|  |  |  |  |  |  | **Maximum range** | **Minimum range** |
| **Umeda River** | － | 659 | 657 | 656 |  | 8800 | 2.5 > |
| **Oono River** | － | － | 17 | 18 |  | 532 | 8 |
| **Shirakawa River** | － | － | － | 2 |  | － | 2.5 > |
| **Tsuboi River** | － | － | － | － |  | 1548 | 2.6 |
