# Supplementary material for: Genetic Diversity of Invasive Spartina alterniflora Loisel. (Poaceae) Introduced Unintentionally Into Japan and Its Invasion Pathway
Source: Front Plant Sci. 2020 Sep 7;11:556039. doi: 10.3389/fpls.2020.556039 (PMC7503347; doi:10.3389/fpls.2020.556039)
Supplement: Supplementary file 2 [file Table_2.docx]

**Supplementary TABLE 2.** The number of *Spartina alterniflora* individuals sampled in each studied river of Aichi and Kumamoto Prefectures and their genotypes.

| **ID** | **Prefecture** | **River** | **Microsatellite markers of *Spartina alterniflora*** | | | | | | | | | | | | | | | | | | | | | | **Sampling**  **year** | **Latitude** | **Longitude** |
| --- | --- | --- | --- | --- | --- | --- | --- | --- | --- | --- | --- | --- | --- | --- | --- | --- | --- | --- | --- | --- | --- | --- | --- | --- | --- | --- | --- |
|  |  |  | **SPR01** | | **SPR02** | | **SPR03** | | **SPR04** | | **SPR05** | | **SPR06** | | **SPR07** | | **SPR08** | | **SPR09** | | **SPR10** | | **SPR11** | |  |  |  |
| AU 1 | Aichi | Umeda | 226 | 226 | 193 | 195 | 313 | 313 | 293 | 293 | 193 | 209 | 238 | 256 | 289 | 289 | 188 | 188 | 269 | 269 | 342 | 348 | 232 | 232 | 2011 | 34°43' 31. 3" | 137°21' 10.6" |
| AU 2 | Aichi | Umeda | 226 | 226 | 193 | 193 | 313 | 313 | 293 | 293 | 193 | 193 | 238 | 238 | 289 | 289 | 180 | 188 | 277 | 277 | 342 | 348 | 232 | 232 | 2011 | 34°43' 31. 3" | 137°21' 10. 6" |
| AU 3 | Aichi | Umeda | 226 | 226 | 193 | 195 | 313 | 313 | 293 | 293 | 193 | 209 | 238 | 256 | 289 | 289 | 188 | 188 | 269 | 269 | 342 | 348 | 232 | 232 | 2011 | 34°43' 31. 0" | 137°21' 10. 6" |
| AU 4 | Aichi | Umeda | 226 | 226 | 195 | 195 | 313 | 313 | 293 | 293 | 193 | 209 | 256 | 256 | 289 | 289 | 188 | 188 | 277 | 277 | 342 | 342 | 232 | 232 | 2012 | 34°40' 44. 6" | 137°17' 10. 6" |
| AU 5 | Aichi | Umeda | 226 | 226 | 193 | 195 | 313 | 313 | 293 | 293 | 193 | 209 | 238 | 256 | 289 | 289 | 188 | 188 | 277 | 277 | 342 | 348 | 232 | 246 | 2012 | 34°43' 23. 8" | 137°21' 02. 1" |
| AU 6 | Aichi | Umeda | 226 | 226 | 193 | 193 | 313 | 313 | 293 | 293 | 193 | 209 | 256 | 256 | 289 | 289 | 180 | 180 | 277 | 277 | 348 | 348 | 232 | 232 | 2012 | 34°42' 29. 5" | 137°20' 09. 8" |
| AU 7 | Aichi | Umeda | 226 | 226 | 195 | 195 | 313 | 313 | 293 | 293 | 193 | 209 | 256 | 256 | 289 | 289 | 180 | 188 | 269 | 277 | 342 | 348 | 232 | 232 | 2012 | 34°43' 10. 9" | 137°20' 31. 8" |
| AU 8 | Aichi | Umeda | 226 | 226 | 195 | 195 | 313 | 313 | 293 | 293 | 193 | 209 | 256 | 256 | 289 | 289 | 180 | 188 | 269 | 269 | 348 | 348 | 232 | 246 | 2012 | 34°42' 50. 3" | 137°20' 19. 6" |
| AU 9 | Aichi | Umeda | 226 | 226 | 195 | 195 | 313 | 313 | 293 | 293 | 193 | 209 | 238 | 238 | 289 | 289 | 188 | 188 | 277 | 277 | 342 | 342 | 232 | 246 | 2012 | 34°41' 38. 4" | 137°19' 20. 0" |
| AU 10 | Aichi | Umeda | 226 | 226 | 193 | 193 | 313 | 313 | 293 | 293 | 193 | 209 | 256 | 256 | 289 | 289 | 188 | 188 | 269 | 269 | 348 | 348 | 232 | 232 | 2012 | 34°43' 31. 0" | 137°21' 10. 6" |
| AU 11 | Aichi | Umeda | 226 | 226 | 195 | 195 | 313 | 313 | 293 | 293 | 193 | 209 | 238 | 256 | 289 | 289 | 180 | 188 | 277 | 277 | 342 | 348 | 232 | 232 | 2012 | 34°42' 27. 3" | 137°20' 09. 1" |
| AU 12 | Aichi | Umeda | 226 | 226 | 195 | 195 | 313 | 313 | 293 | 293 | 193 | 209 | 238 | 256 | 289 | 289 | 180 | 180 | 269 | 277 | 342 | 342 | 232 | 246 | 2012 | 34°43' 23. 8" | 137°21' 02. 1" |
| AU 13 | Aichi | Umeda | 226 | 226 | 195 | 195 | 313 | 313 | 293 | 293 | 193 | 209 | 238 | 256 | 289 | 289 | 180 | 188 | 277 | 277 | 342 | 348 | 232 | 246 | 2012 | 34°42' 29. 5" | 137°20' 09. 8" |
| AU 14 | Aichi | Umeda | 226 | 226 | 193 | 195 | 313 | 313 | 293 | 293 | 193 | 209 | 238 | 238 | 289 | 289 | 188 | 188 | 277 | 277 | 342 | 348 | 232 | 232 | 2013 | 34°43' 16. 4" | 137°20' 35. 2" |
| AU 15 | Aichi | Umeda | 226 | 226 | 195 | 195 | 313 | 313 | 293 | 293 | 193 | 209 | 238 | 238 | 289 | 289 | 188 | 188 | 269 | 277 | 342 | 342 | 232 | 246 | 2015 | 34°42' 53. 8" | 137°20' 21. 7" |
| AU 16 | Aichi | Umeda | 226 | 226 | 195 | 195 | 313 | 313 | 293 | 293 | 193 | 209 | 238 | 238 | 289 | 289 | 188 | 188 | 269 | 277 | 342 | 342 | 232 | 246 | 2015 | 34°42' 54. 0" | 137°20' 21. 9" |
| AU 17 | Aichi | Umeda | 226 | 226 | 195 | 195 | 313 | 313 | 293 | 293 | 193 | 209 | 256 | 256 | 289 | 289 | 180 | 180 | 277 | 277 | 342 | 342 | 232 | 246 | 2015 | 34°42' 18. 3" | 137°20' 02. 4" |
| AU 18 | Aichi | Umeda | 226 | 226 | 193 | 195 | 313 | 313 | 293 | 293 | 193 | 209 | 238 | 238 | 289 | 289 | 180 | 180 | 269 | 277 | 342 | 348 | 232 | 232 | 2015 | 34°42' 47. 1" | 137°20' 18. 0" |
| AU 19 | Aichi | Umeda | 226 | 226 | 193 | 195 | 313 | 313 | 293 | 293 | 193 | 209 | 238 | 256 | 289 | 289 | 180 | 188 | 269 | 277 | 342 | 342 | 232 | 232 | 2015 | 34°42' 52. 1" | 137°20' 20. 9" |
| AU 20 | Aichi | Umeda | 226 | 226 | 193 | 195 | 313 | 313 | 293 | 293 | 193 | 209 | 238 | 256 | 289 | 289 | 180 | 188 | 269 | 277 | 342 | 342 | 232 | 232 | 2015 | 34°42' 52. 7" | 137°20' 21. 3" |
| AU 21 | Aichi | Umeda | 226 | 226 | 195 | 195 | 313 | 313 | 293 | 293 | 193 | 209 | 256 | 256 | 289 | 289 | 180 | 180 | 277 | 277 | 342 | 342 | 232 | 232 | 2015 | 34°42' 59. 3" | 137°22' 02. 7" |
| AU 22 | Aichi | Umeda | 226 | 226 | 193 | 193 | 313 | 313 | 293 | 293 | 193 | 209 | 256 | 256 | 289 | 289 | 180 | 188 | 277 | 277 | 342 | 342 | 232 | 246 | 2015 | 34°42' 58. 8" | 137°22' 05. 2" |
| AU 23 | Aichi | Umeda | 226 | 226 | 193 | 195 | 313 | 313 | 293 | 293 | 193 | 209 | 238 | 256 | 289 | 289 | 180 | 180 | 269 | 277 | 348 | 348 | 232 | 246 | 2015 | 34°42' 58. 2" | 137°22' 07. 6" |
| AU 24 | Aichi | Umeda | 226 | 226 | 193 | 195 | 313 | 313 | 293 | 293 | 193 | 209 | 256 | 256 | 289 | 289 | 188 | 188 | 269 | 277 | 342 | 348 | 232 | 246 | 2015 | 34°42' 57. 7" | 137°22' 10. 1" |
| AU 25 | Aichi | Umeda | 226 | 226 | 195 | 195 | 313 | 313 | 293 | 293 | 193 | 209 | 238 | 256 | 289 | 289 | 180 | 188 | 269 | 269 | 342 | 348 | 232 | 246 | 2015 | 34°42' 57. 1" | 137°22' 12. 6" |
| AU 26 | Aichi | Umeda | 226 | 226 | 193 | 193 | 313 | 313 | 293 | 293 | 193 | 209 | 256 | 256 | 289 | 289 | 180 | 188 | 269 | 277 | 342 | 348 | 246 | 246 | 2015 | 34°42' 56. 5" | 137°22' 15. 1" |
| AU 27 | Aichi | Umeda | 226 | 226 | 193 | 193 | 313 | 313 | 293 | 293 | 193 | 209 | 256 | 256 | 289 | 289 | 188 | 188 | 269 | 269 | 342 | 348 | 246 | 246 | 2015 | 34°42' 56. 0" | 137°22' 17. 6" |
| KO 1 | Kumamoto | Oono | 214 | 214 | 193 | 193 | 313 | 313 | 293 | 293 | 197 | 197 | 253 | 253 | 291 | 295 | 188 | 188 | 269 | 269 | 342 | 348 | 232 | 232 | 2016 | 32°37' 39. 0" | 130°39' 12. 6" |
| KO 2 | Kumamoto | Oono | 214 | 214 | 193 | 193 | 313 | 313 | 293 | 293 | 197 | 197 | 253 | 255 | 291 | 295 | 188 | 188 | 269 | 269 | 342 | 348 | 232 | 246 | 2016 | 32°37' 39. 7" | 130°39' 12. 8" |
| KO 3 | Kumamoto | Oono | 214 | 214 | 193 | 195 | 313 | 313 | 290 | 290 | 197 | 197 | 253 | 255 | 291 | 291 | 180 | 180 | 269 | 269 | 342 | 348 | 246 | 246 | 2016 | 32°37' 46. 5" | 130°39' 16. 0" |
| KO 4 | Kumamoto | Oono | 214 | 214 | 193 | 193 | 313 | 313 | 290 | 290 | 193 | 197 | 253 | 253 | 295 | 295 | 180 | 180 | 269 | 269 | 348 | 348 | 232 | 232 | 2016 | 32°37' 46. 2" | 130°39' 16. 8" |
| KO 5 | Kumamoto | Oono | 214 | 214 | 195 | 195 | 313 | 313 | 293 | 293 | 193 | 197 | 253 | 253 | 291 | 295 | 180 | 180 | 269 | 269 | 342 | 342 | 232 | 232 | 2016 | 32°37' 46. 6" | 130°39' 18. 1" |
| KO 6 | Kumamoto | Oono | 214 | 214 | 193 | 195 | 313 | 313 | 290 | 290 | 193 | 197 | 253 | 255 | 291 | 291 | 180 | 180 | 269 | 269 | 342 | 348 | 232 | 246 | 2016 | 32°37' 46. 7" | 130°39' 18. 5" |
| KO 7 | Kumamoto | Oono | 214 | 214 | 195 | 195 | 313 | 313 | 290 | 290 | 193 | 197 | 253 | 255 | 291 | 295 | 188 | 188 | 269 | 269 | 348 | 348 | 246 | 246 | 2016 | 32°37' 47. 5" | 130°39' 20. 7" |
| KO 8 | Kumamoto | Oono | 214 | 214 | 195 | 195 | 313 | 313 | 290 | 290 | 193 | 197 | 253 | 255 | 291 | 291 | 188 | 188 | 269 | 269 | 348 | 348 | 246 | 246 | 2016 | 32°37' 48. 0" | 130°39' 22. 0" |
| KO 9 | Kumamoto | Oono | 214 | 214 | 195 | 195 | 313 | 313 | 293 | 293 | 197 | 197 | 253 | 255 | 291 | 291 | 180 | 188 | 269 | 269 | 342 | 342 | 232 | 246 | 2016 | 32°37' 48. 7" | 130°39' 24. 3" |
| KO 10 | Kumamoto | Oono | 214 | 214 | 195 | 195 | 313 | 313 | 293 | 293 | 197 | 197 | 253 | 255 | 291 | 295 | 180 | 180 | 269 | 269 | 342 | 342 | 232 | 246 | 2016 | 32°37' 48. 9" | 130°39' 24. 9" |
| KO 11 | Kumamoto | Oono | 214 | 214 | 193 | 195 | 313 | 313 | 290 | 290 | 193 | 193 | 253 | 255 | 291 | 291 | 188 | 188 | 269 | 269 | 342 | 342 | 232 | 232 | 2016 | 32°37' 50. 2" | 130°39' 24. 0" |
| KO 12 | Kumamoto | Oono | 214 | 214 | 193 | 193 | 313 | 313 | 290 | 290 | 197 | 197 | 253 | 255 | 291 | 295 | 180 | 188 | 269 | 269 | 342 | 348 | 232 | 232 | 2016 | 32°37' 49. 9" | 130°39' 24. 3" |
| KO 13 | Kumamoto | Oono | 214 | 214 | 193 | 195 | 313 | 313 | 293 | 293 | 193 | 197 | 253 | 255 | 291 | 291 | 180 | 180 | 269 | 269 | 348 | 348 | 246 | 246 | 2016 | 32°37' 49. 4" | 130°39' 26. 4" |
| KO 14 | Kumamoto | Oono | 214 | 214 | 195 | 195 | 313 | 313 | 293 | 293 | 197 | 197 | 255 | 255 | 291 | 291 | 180 | 180 | 269 | 269 | 342 | 342 | 232 | 246 | 2016 | 32°37' 49. 6" | 130°39' 26. 7" |
| KO 15 | Kumamoto | Oono | 214 | 214 | 193 | 195 | 313 | 313 | 290 | 290 | 193 | 193 | 253 | 255 | 291 | 295 | 180 | 188 | 269 | 269 | 342 | 348 | 232 | 246 | 2016 | 32°37' 49. 7" | 130°39' 27. 0" |
| KO 16 | Kumamoto | Oono | 214 | 214 | 195 | 195 | 313 | 313 | 290 | 290 | 197 | 197 | 255 | 255 | 291 | 291 | 180 | 188 | 269 | 269 | 342 | 348 | 232 | 246 | 2016 | 32°37' 49. 9" | 130°39' 27. 6" |
| KO 17 | Kumamoto | Oono | 214 | 214 | 193 | 195 | 313 | 313 | 290 | 290 | 193 | 197 | 253 | 255 | 291 | 291 | 180 | 188 | 269 | 269 | 342 | 348 | 246 | 246 | 2016 | 32°37' 50. 8" | 130°39' 27. 0" |
| KO 18 | Kumamoto | Oono | 214 | 214 | 193 | 193 | 313 | 313 | 290 | 290 | 193 | 197 | 253 | 255 | 295 | 295 | 188 | 188 | 269 | 269 | 342 | 342 | 232 | 246 | 2016 | 32°37' 51. 3" | 130°39' 29. 8" |
| KO 19 | Kumamoto | Oono | 214 | 214 | 195 | 195 | 313 | 313 | 290 | 290 | 197 | 197 | 253 | 255 | 291 | 291 | 180 | 180 | 269 | 269 | 342 | 342 | 232 | 232 | 2016 | 32°38' 25. 8" | 130°40' 00. 6" |
| KS 1 | Kumamoto | Shirakawa | 204 | 204 | 195 | 195 | 313 | 313 | 326 | 326 | 191 | 197 | 253 | 253 | 287 | 287 | 180 | 180 | 273 | 273 | 344 | 344 | 244 | 246 | 2016 | 32°46' 28. 3" | 130°37' 07. 4" |
| KS 2 | Kumamoto | Shirakawa | 204 | 204 | 195 | 195 | 313 | 313 | 326 | 326 | 191 | 197 | 253 | 253 | 287 | 287 | 180 | 180 | 273 | 273 | 344 | 344 | 244 | 246 | 2016 | 32°46' 28. 3" | 130°37' 07. 4" |
| KS 3 | Kumamoto | Shirakawa | 204 | 204 | 195 | 195 | 313 | 313 | 326 | 326 | 191 | 197 | 253 | 253 | 287 | 287 | 180 | 180 | 273 | 273 | 344 | 344 | 244 | 246 | 2016 | 32°46' 28. 3" | 130°37' 07. 4" |
| KT 1 | Kumamoto | Tsuboi | 204 | 204 | 193 | 197 | 313 | 313 | 326 | 326 | 193 | 197 | 235 | 253 | 287 | 287 | 180 | 180 | 281 | 281 | 344 | 344 | 246 | 246 | 2016 | 32°46' 37. 9" | 130°38' 23. 1" |
| KT 2 | Kumamoto | Tsuboi | 204 | 204 | 193 | 197 | 313 | 313 | 326 | 326 | 191 | 197 | 235 | 253 | 287 | 287 | 180 | 180 | 273 | 281 | 344 | 344 | 246 | 246 | 2016 | 32°46' 37. 5" | 130°38' 22. 7" |
| KT 3 | Kumamoto | Tsuboi | 204 | 204 | 193 | 197 | 313 | 313 | 326 | 326 | 197 | 197 | 235 | 253 | 287 | 287 | 180 | 180 | 281 | 281 | 344 | 344 | 246 | 246 | 2016 | 32°46' 37. 4" | 130°38' 22. 6" |
| KT 4 | Kumamoto | Tsuboi | 204 | 204 | 193 | 193 | 313 | 313 | 326 | 326 | 197 | 197 | 253 | 253 | 287 | 287 | 180 | 180 | 273 | 273 | 344 | 344 | 246 | 246 | 2016 | 32°46' 29. 7" | 130°38' 01. 7" |
| KT 5 | Kumamoto | Tsuboi | 204 | 204 | 193 | 193 | 313 | 313 | 326 | 326 | 197 | 197 | 253 | 253 | 287 | 287 | 180 | 180 | 273 | 273 | 344 | 344 | 246 | 246 | 2016 | 32°46' 29. 5" | 130°38' 01. 4" |
| KT 6 | Kumamoto | Tsuboi | 204 | 204 | 197 | 197 | 313 | 313 | 326 | 326 | 191 | 197 | 235 | 253 | 287 | 287 | 180 | 180 | 281 | 281 | 344 | 344 | 244 | 246 | 2016 | 32°46' 29. 2" | 130°38' 00. 7" |
| KT 7 | Kumamoto | Tsuboi | 204 | 204 | 197 | 197 | 313 | 313 | 326 | 326 | 197 | 197 | 235 | 253 | 287 | 287 | 180 | 180 | 273 | 273 | 344 | 344 | 244 | 244 | 2016 | 32°46' 28. 9" | 130°37' 59. 8" |
| KT 8 | Kumamoto | Tsuboi | 204 | 204 | 193 | 193 | 313 | 313 | 326 | 326 | 191 | 197 | 253 | 253 | 287 | 287 | 180 | 180 | 273 | 281 | 344 | 344 | 244 | 244 | 2016 | 32°46' 28. 8" | 130°37' 58. 5" |
| KT 9 | Kumamoto | Tsuboi | 204 | 204 | 193 | 193 | 313 | 313 | 326 | 326 | 191 | 197 | 253 | 253 | 287 | 287 | 180 | 180 | 273 | 281 | 344 | 344 | 244 | 246 | 2016 | 32°46' 31. 2" | 130°37' 46. 4" |
| KT 10 | Kumamoto | Tsuboi | 204 | 204 | 193 | 197 | 313 | 313 | 326 | 326 | 191 | 191 | 253 | 253 | 287 | 287 | 180 | 180 | 273 | 281 | 344 | 344 | 244 | 244 | 2016 | 32°46' 31. 3" | 130°37' 46. 5" |
| KT 11 | Kumamoto | Tsuboi | 204 | 204 | 193 | 193 | 313 | 313 | 326 | 326 | 191 | 191 | 235 | 253 | 287 | 287 | 180 | 180 | 281 | 281 | 344 | 344 | 244 | 246 | 2016 | 32°46' 31. 3" | 130°37' 44. 5" |
| KT 12 | Kumamoto | Tsuboi | 204 | 204 | 197 | 197 | 313 | 313 | 326 | 326 | 191 | 191 | 253 | 253 | 287 | 287 | 180 | 180 | 273 | 281 | 344 | 344 | 244 | 244 | 2016 | 32°46' 32. 7" | 130°37' 41. 5" |
| KT 13 | Kumamoto | Tsuboi | 204 | 204 | 193 | 193 | 313 | 313 | 326 | 326 | 191 | 191 | 253 | 253 | 287 | 287 | 180 | 180 | 273 | 273 | 344 | 344 | 244 | 246 | 2016 | 32°46' 32. 7" | 130°37' 41. 4" |
| KT 14 | Kumamoto | Tsuboi | 204 | 204 | 193 | 193 | 313 | 313 | 326 | 326 | 191 | 191 | 253 | 253 | 287 | 287 | 180 | 180 | 273 | 273 | 344 | 344 | 246 | 246 | 2016 | 32°46' 40. 5" | 130°37' 32. 9" |
| KT 15 | Kumamoto | Tsuboi | 204 | 204 | 193 | 193 | 313 | 313 | 326 | 326 | 191 | 191 | 253 | 253 | 287 | 287 | 180 | 180 | 273 | 273 | 344 | 344 | 244 | 246 | 2016 | 32°46' 40. 5" | 130°37' 32. 6" |
| KT 16 | Kumamoto | Tsuboi | 204 | 204 | 193 | 193 | 313 | 313 | 326 | 326 | 191 | 191 | 253 | 253 | 287 | 287 | 180 | 180 | 273 | 273 | 344 | 344 | 244 | 246 | 2016 | 32°46' 40. 5" | 130°37' 31. 9" |
| KT 17 | Kumamoto | Tsuboi | 204 | 204 | 193 | 193 | 313 | 313 | 326 | 326 | 191 | 191 | 253 | 253 | 287 | 287 | 180 | 180 | 273 | 273 | 344 | 344 | 244 | 244 | 2016 | 32°46' 40. 5" | 130°37' 31. 3" |
| KT 18 | Kumamoto | Tsuboi | 204 | 204 | 193 | 193 | 313 | 313 | 326 | 326 | 191 | 191 | 253 | 253 | 287 | 287 | 180 | 180 | 273 | 273 | 344 | 344 | 244 | 246 | 2016 | 32°46' 40. 5" | 130°37' 29. 7" |
| KT 19 | Kumamoto | Tsuboi | 204 | 204 | 193 | 193 | 313 | 313 | 326 | 326 | 191 | 197 | 253 | 253 | 287 | 287 | 180 | 180 | 281 | 281 | 344 | 344 | 246 | 246 | 2016 | 32°46' 40. 6" | 130°37' 28. 4" |
| KT 20 | Kumamoto | Tsuboi | 204 | 204 | 193 | 193 | 313 | 313 | 326 | 326 | 191 | 197 | 235 | 253 | 287 | 287 | 180 | 180 | 273 | 273 | 344 | 344 | 244 | 246 | 2016 | 32°46' 42. 0" | 130°37' 23. 8" |
